# Biostimulant Potential of Acetic Acid Under Drought Stress Is Confounded by pH-Dependent Root Growth Inhibition

**DOI:** 10.3389/fpls.2020.00647

**Published:** 2020-05-25

**Authors:** Megan M. Allen, Damian J. Allen

**Affiliations:** ^1^School of Agriculture, Policy and Development, University of Reading, Reading, United Kingdom; ^2^Department of Agronomy, Purdue University, West Lafayette, IN, United States

**Keywords:** drought, *Zea mays*, roots, maize, acetate, acetic acid, water use efficiency

## Abstract

Recent reports of acetic acid-induced drought tolerance and avoidance across a diverse range of plant species encourage consideration of this low-cost commodity organic acid as a biostimulant. These results are surprising as they contrast with earlier studies showing pH-dependent root growth inhibition at similar concentrations. We test the hypothesis that the concentration of the membrane permeable undissociated form of acetic acid (CH_3_COOH) selectively inhibits maize root growth, and subsequently evaluate its impact on seedling water use and growth under deficit irrigation. We demonstrate conclusively for the first time that when germinating maize on filter paper, low pH exacerbates, and high pH mitigates, this inhibition of root growth in a predictable manner based on the dissociation constant of acetic acid. The buffering capacity of potting media can reduce this root damage through keeping the acetic acid primarily in the membrane impermeable dissociated form (CH_3_COO^–^) at near neutral pH, but peat substrates appear to offer some protection, even at low pH. While both deficit irrigation and acetic acid reduced water use and growth of maize seedlings outdoors, there was no significant interaction between the treatments. Twenty nine millimolar total acetic acid (CH_3_COOH + CH_3_COO^–^) reduced transpiration, compared to lower and higher concentrations, but this did not specifically improve performance under reduced water availability, with parallel declines in shoot biomass leading to relatively consistent water use efficiency. Any acetic acid biostimulant claims under water stress should characterize its dissociation level, and exclude root damage as a primary cause.

## Introduction

A low dose (<50 mM) of acetic acid has recently been proposed as a biostimulant under drought stress for major crops as diverse as maize (*Zea mays* L.) (Kim et al., 2017), cassava (*Manihot esculenta* Crantz) (Utsumi et al., 2019), and mung bean (*Vigna radiata* L.) (Rahman et al., 2019). Acetic acid could bypass many of the barriers to commercialization of new biostimulants (Yakhin et al., 2017), as a well-studied compound with low cost (1 US$ per kL of 50 mM, based on 330 US$ per t; ICIS Chemical News, 2020) scaled-up production (12 million t/year; Le Berre et al., 2014) through industrial and food use, with known toxicology (Le Berre et al., 2014) and even regulatory approval as an organic herbicide at higher doses (20%; US EPA, 2019). The biostimulation is surprising as both drought and acetic acid have been ubiquitous throughout plant evolution and crop domestication, and so the key supportive prior observations are summarized in the next paragraph, but note they were all conducted in controlled environments.

Root growth in 5-day-old barley (*Hordeum vulgare* L.) seedlings was increased when germinated in 0.1–1 mM acetic acid solutions at a pH of 6.5 (Lynch, 1977). In greenhouse-grown maize, 10-day-old seedlings were treated with 0–50 mM of acetic acid through the growth media for 4 days, water was withheld for 6 days and then re-watered for 5 days, with survival significantly higher after application of 30 mM acetic acid compared to the control (Kim et al., 2017). A significant increase in above ground biomass, leaf area, leaf total chlorophyll content, instantaneous leaf-level Water Use Efficiency (WUE), leaf temperature and shoot Relative Water Content (RWC) was observed in response to foliar application of 20 mM acetic acid to 16-day-old potted mung beans every 2 days for 2 weeks, with a significant reduction in leaf transpiration rate (Rahman et al., 2019). When this foliar acetic acid application was combined with saline growth media irrigation, in addition to all these impacts, acetic acid also significantly increased root biomass, root length and leaf carbon assimilation rate, compared to the foliar water sprayed controls with the same saline growth media irrigation (Rahman et al., 2019). In greenhouse-grown cassava, treatment with 10 mM acetic acid for 1 week enhanced drought avoidance during water withholding for the subsequent 2 weeks, as demonstrated by reduced wilting, higher leaf RWC, higher chlorophyll content, higher leaf temperature, and lower transpiration, compared to droughted controls (Utsumi et al., 2019).

Alongside these positive impacts, the prior studies identified dosing complexities which need to be addressed before acetic acid can be applied as a biostimulant to commercial maize production. The improvements observed with 30 mM acetic acid in maize were not significant at either lower (10 and 20 mM) or higher (50 mM) doses (Kim et al., 2017). In contrast to the protection from drought-induced wilting seen with 10 mM, 20–50 mM acetic acid applications to cassava actually induced wilting in the absence of drought (Utsumi et al., 2019). The barley seedling root growth stimulation seen at 0.1–1 mM acetic acid at a pH of 6.5 was eliminated for these same concentrations at a pH of 3.5 and a substantial root inhibition was observed at higher acetic acid concentrations (10 and 50 mM) in barley at a pH of 6.5, but not in maize (17 mM at a pH of 6.4) (Lynch, 1977). Root growth in reed (*Phragmites australis*) was reduced at 0.3 mM, and entirely inhibited at 1.7 mM acetic acid (Armstrong et al., 1996).

As an important food preservative, the mode of action of acetic acid stress on micro-organisms has long been studied. As a weak acid, the concentration of the undissociated ([CH_3_COOH], hereafter referred to as [HAc], Lawford and Rousseau, 1993) form of acetic acid approaches a pH-dependent equilibrium with the concentration of the dissociated form ([CH_3_COO^–^], hereafter referred to as [Ac^–^]) in aqueous solutions. HAc is lipid soluble and therefore can pass across cell membranes, in contrast to the hydrophilic Ac^–^ (Lawford and Rousseau, 1993). Under acidic extracellular conditions, HAc crossing the plasma membrane will have the potential to dissociate to H^+^ and Ac^–^ ions inside the cell as a near neutral intracellular pH is often maintained (Russell, 1992; Warnecke and Gill, 2005). While there has been debate about the relative toxicity of these species inside the cell (Russell, 1992), it’s clear that extracellular HAc is more toxic than extracellular Ac^–^, due to its ability to get into the cells of yeast (Noda et al., 1982) and bacteria (Diez-Gonzalez and Russell, 1997; Warnecke and Gill, 2005), and this mechanism has been proposed to also apply to plants (Armstrong et al., 1996). The equilibrium of [HAc] and [Ac^–^] is shifted significantly within agronomically-relevant pH ranges, and the potential for an interaction between total acetic acid concentration (defined as [HAc] + [Ac^–^]) and pH is mostly absent from the plant literature discussed above, with pH almost never actively controlled and typically not even reported.

Is it possible that a dose- and pH-dependent impact on root growth is the primary response to growth media acetic acid application and that is subsequently responsible for all the phenotypes reported? Here we tested for the first time the hypothesis that it is [HAc], rather than [HAc] + [Ac^–^] that impacts seedling root growth, predicting that for a given applied [HAc] + [Ac^–^] the detrimental effect on roots is alleviated by raising the pH and exacerbated by lowering the pH in a predictable manner based on the known dissociation constant in aqueous solutions. Secondly, we evaluated whether these observations on seedling root growth at different pHs in aqueous solutions translate to germination in growth media with pH buffering capacity. Finally, we applied acetic acid to maize growing in pots outdoors for the first time, imposing a fully factorial [HAc] + [Ac^–^] by deficit irrigation design, and examined impacts on growth and water use, as a necessary step toward the evaluation of acetic acid as a commercial biostimulant in maize.

## Materials and Methods

### Acetic Acid and pH Impacts on Seedlings

All experiments were undertaken in a domestic environment, therefore chemicals were selected based on local availability and regulatory approval for consumer use. Acetic acid treatments (0, 0.01, 0.1, 1, 10, 20, 29, 38, 47, and 100 mM of [HAc] + [Ac^–^]) were prepared from food-grade acetic acid (Heinz All Natural Distilled White Vinegar 5% acidity, Kraft Heinz Foods, Pittsburgh, PA, United States), diluted with municipal drinking water. This 5.23% acetic acid (weight%) was fermented from distilled maize ethanol, and also contained 0.20% residual ethanol, 0.02% ethyl acetate with < 0.01% other organic acids as determined by ^1^H quantitative NMR (John Edwards, Pers. Comm.). The simplicity of this industrial vinegar (Gerbi et al., 1997; Sáiz-Abajo et al., 2005; Mas et al., 2016; Schneider et al., 2018) strongly contrasts with the diversity of composition of fruit- and wine-derived traditional vinegars (Caligiania et al., 2007; Edwards, 2018). 31% HCl (Crown^®^ Muriatic Acid, Packaging Service, Pearland, TX) and 18–28% NaOH with 0–1% KOH (Instant Power^®^ Hair Clog Remover, Scotch, Dallas, TX) were used to develop 10 aliquots of each of the 10 [HAc] + [Ac^–^] spanning a broad range of pH (>2 and <13), measured before and after seedling growth with a LAQUAtwin pH-33 (Horiba, Irvine, CA, United States) sensor. [HAc] was estimated based on acetic acid stock, measured pH value and an acid dissociation constant (K_*a*_) of 1.8 × 10^–5^ at 25°C (Lawford and Rousseau, 1993). Creped seed germination paper (0.25 × 0.38 m, SB39211, Nasco, Fort Atkinson, WI, United States) was moistened with the pH-adjusted acetic acid solution and then 6 fungicide-treated maize seeds (Hybrid 1156, Steyer Seeds, Tiffin, OH, United States) were spaced evenly 5 cm from the long edge of the paper, rolled tightly, secured with a rubber band below the seeds, placed in a 0.03 × 0.2 m (diameter × length) 110 ml glass test tube with the seed at the top of the tube and filled with additional pH-adjusted acetic acid solution. These 100 test tubes were completely randomized in racks and placed under plant growth lights (GLP24FS/19W/LED, Feit Electric, Pico Rivera, CA, United States) producing ∼200 μmol m^–2^ s^–1^ photosynthetically active radiation (PAR) at the top of the paper rolls, as measured with a quantum sensor (LGBQM, Hydrofarm, Petaluma, CA, United States), with a 16/8 photoperiod. After 4 days of growth at room temperature, the maximum root and shoot length of each seedling was recorded. While study of primary root and coleoptile length is an established high throughput technique to monitor impacts on growth (Pace et al., 2014), in this study we did not attempt to establish a correlation with seedling root and shoot biomass. Mean (±1 standard deviation) air temperature, relative humidity and vapor pressure deficit (VPD) was 26.4 ± 1.0°C, 54 ± 3% and 1.6 ± 0.2 kPa, respectively, as measured with an adjacent sensor (WH31B, Ambient Weather, Chandler, AZ) logged every 5 min by a weather station (WS-2000, Ambient Weather).

### Acetic Acid and Growth Media Impacts on Seedlings

Seedling root and shoot growth were compared in a factorial experiment of 2 growth media × 6 acetic acid treatments, arranged in a randomized complete block design with 6 replicates. Translucent polypropylene pots (0.95L, S-22771, Uline, Pleasant Prairie, WI, United States) were filled with either higher-pH potting mix (Miracle-Gro Moisture Control Potting Mix, Scotts, Marysville, OH, United States) or lower-pH sphagnum peat moss (Miracle-Gro, Scotts). The peat media contained 99% peat, 0.14% wetting agent, 0.10% ammoniacal nitrogen, 0.09% nitrate nitrogen, 0.11% P_2_O_5_, and 0.15% K_2_O. The potting mix was a proprietary blend of sphagnum peat moss, processed forest products, compost, coir, perlite, wetting agent, 0.11% ammoniacal nitrogen, 0.10% nitrate nitrogen, 0.11% P_2_O_5_, and 0.16% K_2_O. Mean (± 1 standard deviation) electrical conductivity (HI98331 Gro line, Hanna Instruments Inc., Woonsocket, RI) was 1.27 ± 0.13 mS cm^–1^ for potting mix and 1.21 ± 0.05 mS cm^–1^ for peat, after saturation with distilled water. While these were selected due to their contrasting advertised pH (2.8–4.0 for peat and 6.5–7.5 for potting mix) and similar fertilizer contents and conductivities, we cannot exclude the possibility of other differences influencing seedling responses to acetic acid, such as hydraulic properties and microbial communities. Individual pots were brought to 100% growth media RWC by irrigating with 0, 10, 20, 29, 38, or 47 mM [HAc] + [Ac^–^] until they reached the target weight on a calibrated balance (0.1 g precision, SPX2201, Ohaus, Parsippany, NJ, United States). Free-draining growth media after saturation, based on the average of representative pots, defined 100% growth media RWC. No pH adjustment was made to these acetic acid solutions. Single maize seeds were planted 3–4 cm deep on August 26, 2019 and kept at room temperature for 2–3 days until emergence to avoid potential germination inhibiting pot temperatures above 35°C (Andrade et al., 2018), and then they were moved to an outdoor location in Katy, TX (29°42′N 95°50′W) out of direct sunlight. During this experiment, mean (± 1 standard deviation) air temperature, relative humidity and VPD was 25.8 ± 0.2°C, 54 ± 1%, and 1.52 ± 0.03 kPa indoors and 29.3 ± 4.2°C, 72 ± 16%, and 0.53 ± 0.51 kPa outdoors, with no precipitation. Maximum solar radiation measured by the weather station in full sunlight during this period was 928 W m^–2^. PAR was recorded in the shade at ∼2 h intervals throughout a day with a quantum sensor and correlated to solar radiation measured by the weather station, and this was used to estimate an approximate maximum PAR in the shade during the experiment of 230 μmol m^–2^ s^–1^, or 12% of full-sun. Growth media pH was estimated by sampling ∼15 ml of media from all pots in 3 reps during seedling growth in a 150 ml polypropylene cup (B071D8S33H, Tashibox, ASF TASHI LLC, Pittsfield, MA), adding 2.5 times its weight in distilled water, incubated for 30 mins on an orbital shaker (COZOORKJBDUS, Amazon, Seattle, WA) at 100 rpm before measuring the pH of the solution. Root and shoot length was measured 4 days after planting. As in the prior experiment we did not attempt to establish a correlation with seedling root and shoot biomass. Statistix version 9.0 (Analytical Software, Tallahassee, FL) was used for a blocked factorial analysis of variance, followed by a Tukey all pairwise comparison (α = 0.05).

### Acetic Acid and Drought Impacts on WUE

**FIGURE 1:**
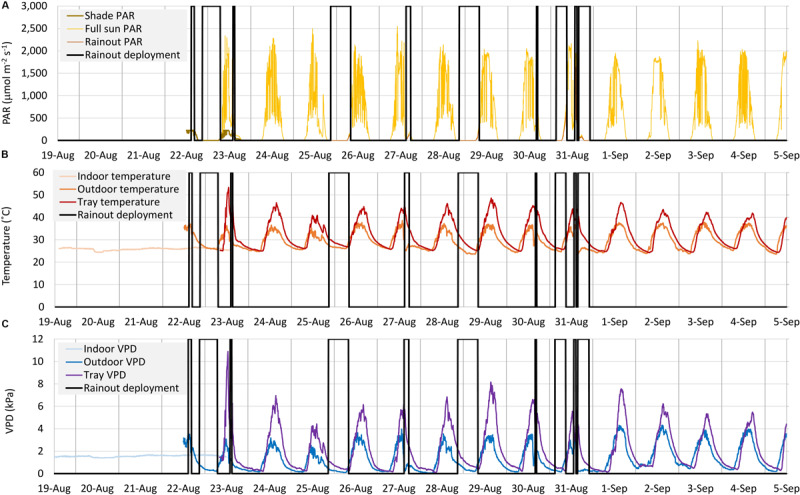
Time course for Photosynthetically Active Radiation (PAR) **(A)**, Temperature **(B)** and Vapor Pressure Deficit (VPD) **(C)** under indoor, shade (acclimation), full-sun and during rainout shelter deployment phases of the outdoor Water Use Efficiency (WUE) assay. Tray temperature and VPD reflect the average of three sensors adjacent to the plant pots.

A factorial experiment of 4 water × 6 acetic acid treatments was used, arranged in a randomized complete block design with 6 replicates. Pots and acetic acid solutions were prepared as in section Acetic Acid and Growth Media Impacts on Seedlings, except only the higher pH potting mix was used. Planting occurred on August 19, 2019, pots were covered with a low density polyethylene lid with a 2.9 cm diameter hole drilled in the middle, and pots were maintained at room temperature for 3–4 days. With observed tray temperatures in August in southern TX (Figure 1B) beyond the 35–40°C permissible limit for maize germination (Andrade et al., 2018), this indoor-to-outdoor seedling transfer was required. Four water treatments (25, 50, 75, and 100%) were imposed between the extremes of 0 and 100% potting mix RWC, with the former defined after drying representative substrate to a constant weight at ∼77°C in a fan-assisted oven (JKP30SP2SS, General Electric, Louisville, KY, United States). All pots were started at 100% RWC, and then every 1–3 days, depending on the rate of weight loss, each pot was weighed and, as needed, returned to the target weight by watering with the appropriate acetic acid concentration. Pot location within a 24 plant block was re-randomized after each weighing. Similar pots with drilled lids but without seed, and 3 pots which failed to germinate, were used as evaporation controls. Precipitation was excluded by manual deployment of transparent 150 μm polyethylene rainout shelters (3 × 0.6 × 0.5 m, Haxnicks 50–5000, Tierra-Derco, Jasper, IN, United States) when rainfall was forecast. PAR was measured under the rainout shelter at ∼2 h intervals throughout a day with the quantum sensor and correlated to solar radiation measured by the weather station. The rainout shelters, which reduced PAR by about 15%, were deployed for 13% of the outside phase duration of the WUE assay, with more than 60% of this deployment time in the dark (Figure 1A). Due to uncertainty in weather forecasting, not all deployments coincided with measurable rainfall (e.g., night of August 28–29). Midday air temperatures were around 35°C, with tray temperatures, adjacent to the plant pots, above 40°C (Figure 1B). While daytime deployment increased the difference between outside air and tray temperatures (e.g., August 27), this was primarily driven by delaying the precipitation-associated outside air cooling, rather than a greenhouse effect, and peak tray temperatures did not coincide with deployment times (Figure 1B). The observed divergence in VPD between outside air and the trays at these times was associated with higher humidity, as well as these lower temperatures, outside the shelters during the precipitation event, and extreme tray VPD also did not coincide with deployment times (Figure 1C).

Zeta-cypermethrin insecticide (GardenTech Sevin Insect Killer Concentrate, TechPac, Atlanta, GA, United States) was applied at labeled maize rate on August 27. Non-destructive chlorophyll contents were estimated 13 days after planting from absorbance with the atLEAF STD (FT Green, Wilmington, DE) (Zhu et al., 2012) approximately at the midpoint of the youngest leaf with ligule emergence, avoiding the mid vein. Plant mature leaf area 14 days after planting was estimated non-destructively from the width (digital calipers) and length (ruler) of each leaf with ligule emergence, assuming rectangular leaf geometry. Stem (culm) volume 15 days after planting was calculated from stem diameter and stem height, assuming cylindrical geometry. Stem diameter was measured as the maximum width at lid height with digital calipers. Stem height was read with a ruler from the lid to the youngest visible ligule. Vegetative developmental stage was based on the number of leaves with an emerged ligule at harvest 16–17 days after planting. The fresh weight of the biomass above the lid was recorded on the calibrated balance (0.1 g precision), manually cut into ∼2 cm pieces and water content determined on a ∼1.5 g subsample utilizing pre-dried mini cupcake paper cases, oven and a calibrated balance (1 mg precision, USS-DBS15-3, U.S. Solid, Cleveland, OH, United States), to enable estimation of above-ground dry weight. After harvest the area that roots were visible on the bottom of each pot were estimated from images captured by a flatbed scanner (H625cdw, Dell, Round Rock, TX, United States). A grid was pasted over each pot base image in Microsoft Powerpoint and a root was manually scored as present for each of the 314 squares if it covered ≥50% of the square. While root observations at transparent interfaces with growth media are well established techniques (Huck and Taylor, 1982; Smit et al., 2000), in this system we did not relate this non-destructive root area measurement with more physiologically-relevant root phenotypes, such as biomass.

Evapotranspiration rate was calculated as the sum of the loss of weight between irrigations. The hole in the lid will result in some direct evaporation from the growth media that will vary due to both the remaining available water in the pot and the weather conditions during that period. A sigmoidal curve was fit to the relationship between evaporation rate from the unplanted/ungerminated controls and their weight (pot + lid + media + water), separately for each watering interval, using the equation below implemented in the eeFIT (v1.05) Microsoft Excel Add-In (Vivaudou, 2019):

$$\mathrm{Evaporation}\,\mathrm{rate}=\mathrm{Max}\,\frac{W^{h}}{W+K^{h}}$$

Where:

*Evaporation rate* = grams of water loss per day per pot.

*Max* = Maximum evaporation rate; iteratively fit, initiated at 1 g day^–1^.

*W* = Measured weight of pot before re-watering (g).

*h* = Maximum slope; iteratively fit, initiated at 1.

*K* = Weight of pot at 50% of *Max*; iteratively fit, initiated at 1 g.

While *W* included the pot, lid and growth media weight, these were small and relatively consistent compared to the variation in water content between control pots. This estimate of evaporation rate for each day and pot weight was then subtracted from the measured evapotranspiration rate for each plant to estimate daily transpiration rate and this was summed over the experiment and used with above ground biomass to estimate WUE.

## Results

### Acetic Acid and pH Impacts on Seedlings

**FIGURE 2:**
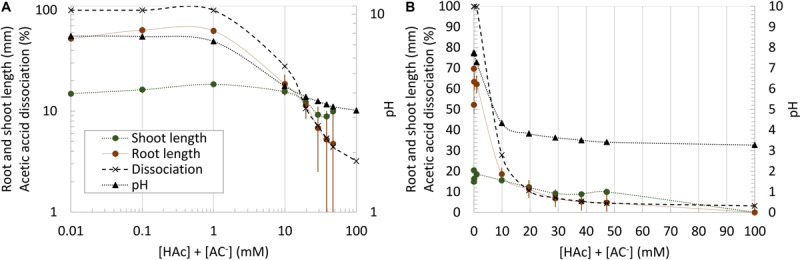
Total (undissociated and dissociated) acetic acid concentration ([HAc] + [Ac^–^]) impacts on seedling growth in germination paper rolls, along with pH and acetic acid dissociation. Mean root and shoot data (±1 standard error; *n* = 6) are plotted both on a logarithmic **(A)** and linear **(B)** scale.

**FIGURE 3:**
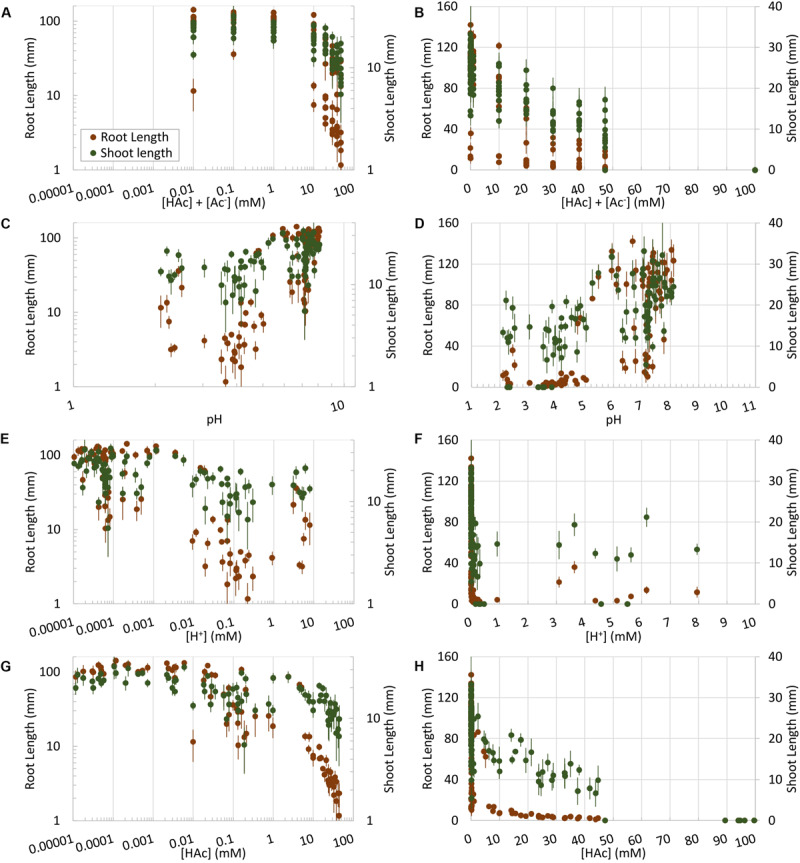
Undissociated acetic acid ([HAc]) **(G,H)** impacts on root and shoot growth on germination paper, along with total acetic acid ([HAc] + [Ac^–^]) **(A,B)** or acidity expressed as [H^+^] **(E,F)** or pH **(C,D)**. Mean root and shoot data (±1 standard error; *n* = 6) are plotted both on logarithmic **(A,C,E,G)** and linear **(B,D,F,H)** scales.

Maize root and shoot growth in unbuffered water-soaked germination paper rolls were significantly (*p* < 0.05) inhibited by acetic acid at doses as low as 10 mM, compared to 0 mM controls, however, this parallels similar drops in pH and acetic acid dissociation over this range (Figure 2). In contrast to Figure 2, when root and shoot growth were examined in rolled germination paper across 100 incubations where pH and acetic acid concentration were uncoupled, the robust trend between [HAc] + [Ac^–^] and seedling growth was lost (Figures 3A,B). However, the root and shoot inhibition was also not simply predicted by pH (Figures 3C,D) or [H^+^] (Figures 3E,F). Rather, shoot and root length was responding tightly to [HAc] (Figures 3G,H). These results in Figures 3C–H are based on pH measured at the end of the experiment, but similar results were obtained when the initial pH was used (data not shown). An estimated [HAc] above 10 mM inhibited root growth by >90%, while shoot growth inhibition didn’t reach this level until >45 mM [HAc]. No robust stimulation of seedling root or shoot growth were observed over 4 orders of magnitude of [HAc] + [Ac^–^] (Figures 3A,B).

### Acetic Acid and Growth Media Impacts on Seedlings

**FIGURE 4:**
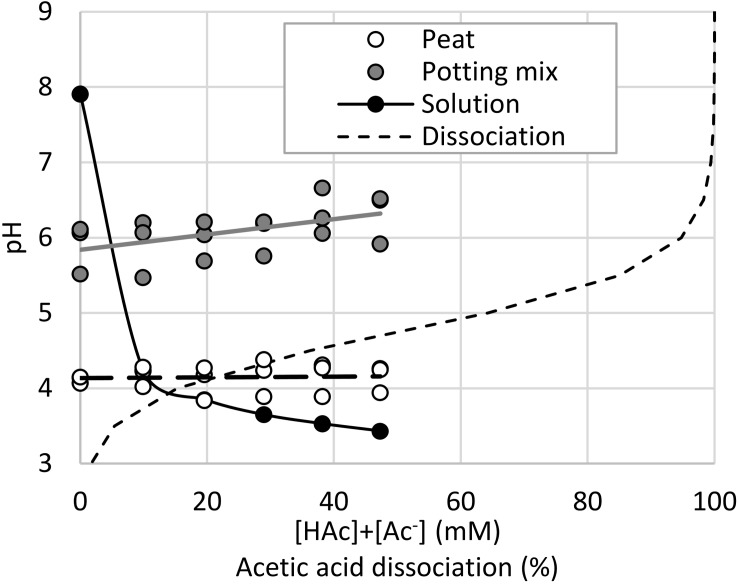
pH of a range of total (undissociated and dissociated) acetic acid ([HAc] + [Ac^–^]) irrigation solutions and the resulting pH of treated seedling peat and potting mix growth media (*n* = 3), fitted with a linear regression, along with the expected dissociation of acetic acid at 25°C.

**FIGURE 5:**
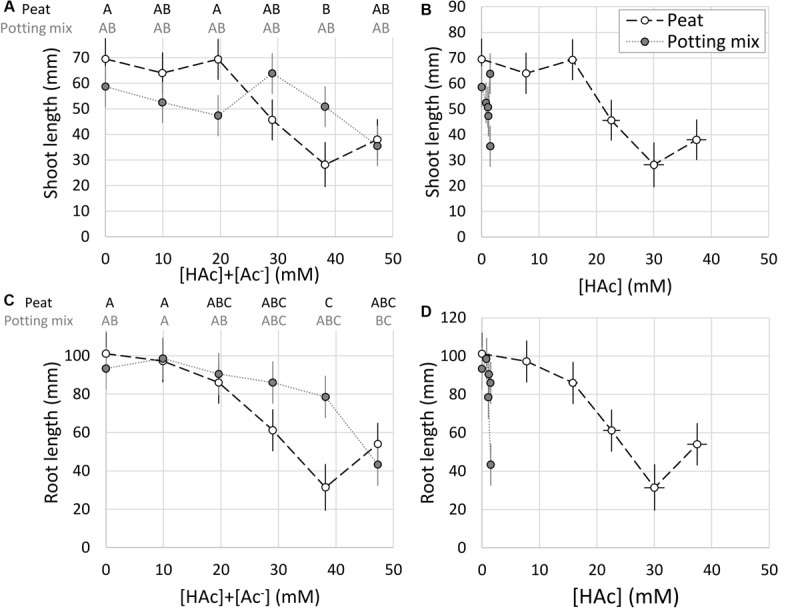
Total ([HAc] + [Ac^–^]) **(A**,**C)** and undissociated ([HAc]) **(B,D)** acetic acid concentration impacts on seedling root **(C,D)** and shoot **(A,B)** growth (mean ± 1 standard error of the mean) in peat and potting mix growth media with contrasting pH. Significant differences between treatments (*p* < 0.05) are marked with contrasting letters (**A**,**C**; blocked factorial ANOVA; Tukey pairwise comparison; *n* = 6).

The peat and potting mix substrates had markedly different pH values, and in contrast to the irrigation solution, increasing acetic acid concentrations did not reduce the pH. Figure 4 demonstrates that with pH values below 4.5 for the irrigation solutions and peat, the acetic acid was almost entirely undissociated. In contrast, the buffering capacity of the potting mix maintained the pH above 6 where the acetic acid was mostly dissociated. Significant acetic acid impacts on seedling shoot and root growth were observed (*p* < 0.05), but not related to the substrate type (*p* > 0.1). Growth media type × acetic acid was weakly significant (*p* = 0.05–0.1), specifically with 38 mM [HAc] + [Ac^–^] significantly (*p* < 0.05) reducing both root and shoot length in peat, compared to the 0 mM controls, whereas in the potting mix 47 mM was required to significantly stunt even the root growth (Figures 5A,C). When expressed on an undissociated basis (Figures 5B,D), significant root and shoot inhibition was detected at 1.2 mM [HAc] in potting mix, but only above 29 mM in peat, and none of these treatments on peat produced the >90% reduction in root length seen on germination paper at these [HAc] (Figures 3G,H). No significant stimulation of seedling root or shoot growth was observed at any acetic acid dose or substrate (Figures 5A,C).

### Acetic Acid and Drought Impacts on WUE

**FIGURE 6:**
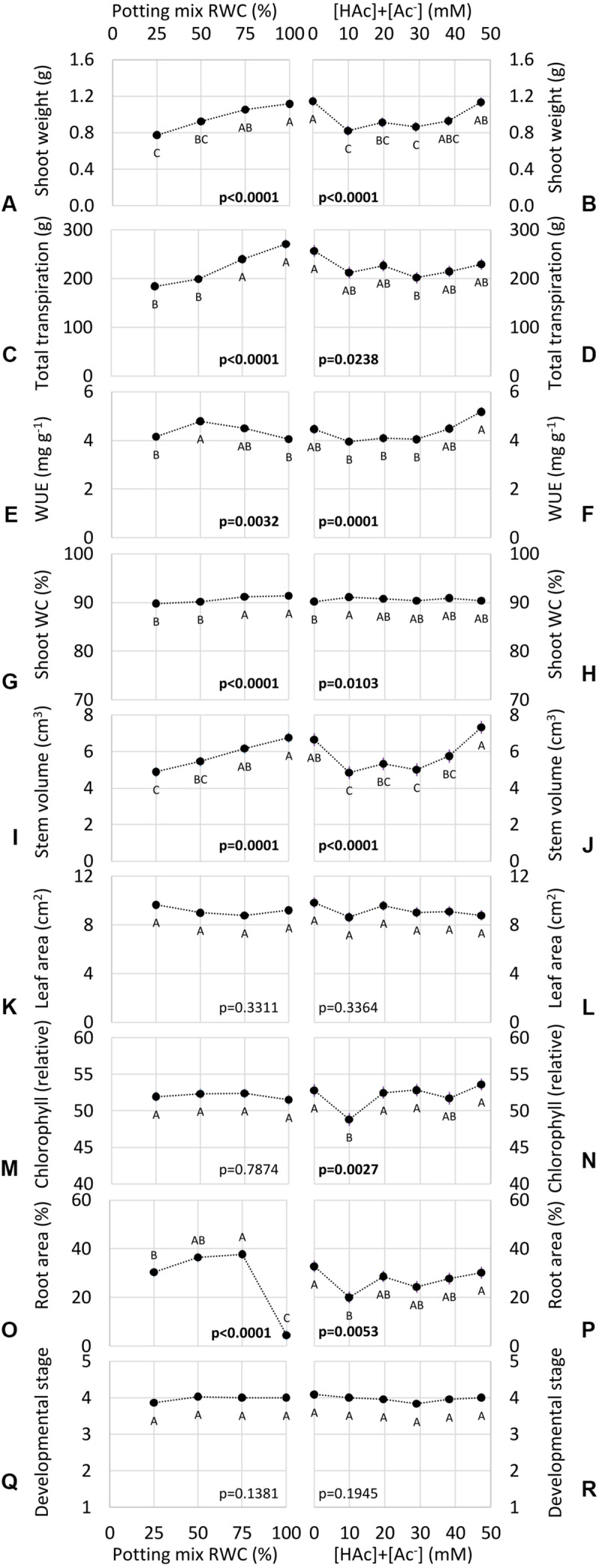
Potting mix Relative Water Content (RWC) and total (undissociated and dissociated) acetic acid ([HAc] + [Ac^–^]) treatment impacts in the outdoor Water Use Efficiency (WUE) assay on a range of seedling growth phenotypes (means ± 1 standard error of the mean), including shoot weight **(A,B)** and Water Content (WC) **(G,H)**, leaf area **(K,L)** and chlorophyll content **(M,N)**, stem volume **(I,J)**, root area **(O,P)** and plant transpiration **(C,D)**, WUE **(E,F)** and developmental stage **(Q,R)**. None of the water by acetic acid interactions were significant (*p* > 0.1), so only the main effects are presented. Contrasting letters denote significant differences (*p* < 0.05) within water **(A,C,E,G,I,K,M,O,Q)** or acetic acid **(B,D,F,H,J,L,N,P,R)** treatments (blocked factorial ANOVA; Tukey pairwise comparison; *n* = 6).

Water and acetic acid treatments impacted seedling growth, but no significant interactions were observed (*p* > 0.1), therefore only main water and acetic acid effects are shown in Figure 6. Clear dose responses to water availability were observed in many of these phenotypes, including shoot weight (Figure 6A), stem volume (Figure 6I), transpiration (Figure 6C), and shoot water content (Figure 6G). Whole plant WUE (Figure 6E) and root area (Figure 6O) exhibited significantly higher values at intermediate water availability. Leaf area (Figure 6K), leaf chlorophyll content (Figure 6M) and developmental stage (Figure 6Q) were not significantly impacted by water treatment.

In contrast to water availability, none of these phenotypes exhibited a straight-forward acetic acid dose response, demonstrated by no significant differences between the lowest (0 mM) and highest (47 mM) [HAc] + [Ac^–^] for any measurement (Figure 6). This is despite 10, 20, and/or 29 mM [HAc] + [Ac^–^] having significantly lower stem weight, stem volume, transpiration, chlorophyll content and root area, than 0 mM (Figure 6). As with the water treatments, no significant acetic acid impacts were observed on leaf area (Figure 6L) or developmental stage (Figure 6R). No growth stimulation was observed at any acetic acid dose (Figure 6).

## Discussion

In contrast to prior observations in barley (0.1–1 mM acetic acid at a pH of 6.5) (Lynch, 1977), no treatment increased root growth on germination paper in maize, despite application of 4 orders of magnitude in acetic acid concentration (Figure 3) and 12 pH units. Rather we confirmed our prediction that lowering the pH (e.g., <3) of low acetic acid doses (e.g., 10 mM) increased damage to roots, and raising the pH (e.g., >5) of high doses of acetic acid (e.g., 20 mM) protected roots growing on poorly buffered germination paper, in a predictable manner based on the known dissociation constant of this weak acid. This supports our hypothesis that [HAc] is causing seedling growth inhibition responses on germination paper, rather than [HAc] + [Ac^–^], with greater sensitivity of root than shoot growth (Figure 3). High pH protection of high acetic acid concentrations is not complete, suggesting additional detrimental factors are at play under these conditions. This root growth sensitivity complicates interpretation of the recently reported water stress related phenotypes induced by acetic acid (Kim et al., 2017; Isaji et al., 2018; Rahman et al., 2019; Utsumi et al., 2019), where [HAc] was undefined. The presence of low concentrations of residual ethanol in the treatment vinegar has the potential to confound these acetic acid effects. While this cannot be entirely discounted from the available data, significant treatment impacts were observed at 10 mM acetic acid, where ethanol concentrations would be <0.4 mM, that is more than two orders of magnitude below the ethanol treatment with observed impacts on abiotic stress tolerance in *Arabidopsis* and rice (*Oryza sativa* L.) (Kato-Noguchi, 2008; Nguyen et al., 2017). In contrast to weak acids like acetic, there is also not an obvious mechanism for the observed interaction between ethanol concentration and pH.

The organic components of the potting media, especially the peat present in both, contribute significant buffering capacity (Blok et al., 2017) which eliminated any reduction in pH from acetic acid addition, uncoupling acetic acid concentration and expected dissociation, in contrast to unbuffered solutions (Figure 4). Figure 5 is consistent with the prior observation that high acetic acid stunts seedling growth (Figure 3), but this is mostly ameliorated by the high pH (∼6) and buffering capacity of the potting mix. While this inhibition was apparent at lower acetic acid concentrations when grown in the lower pH (∼4) peat, this was not as large an impact as expected, considering almost all the acetic acid is expected to be undissociated at this pH. This suggests that peat provides additional protection of roots from high [HAc] damage, compared to rolled germination paper, such as through increased macronutrient availability. Intriguingly, with peat-derived (Billard et al., 2014) humic substances also identified as biostimulants in maize (Savy et al., 2020), there is the potential for an indirect impact of acetic acid on seedlings through production and release of more active humic compounds from the growth media. With acetic acid a known substrate of soil bacteria and fungi, particularly under aerobic conditions (Sigren et al., 1997; Chauhan and Ogram, 2006; Ostendorf et al., 2007; Herron et al., 2009), microbial community and oxygen availability are also potential interacting factors for additional study, along with testing in media only differing in buffering capacity and more diverse and agronomically-relevant soils. While these seedling experiments were helpful in understanding the interaction between root growth, acetic acid and pH, they were all conducted under well hydrated conditions, and so may not relate to the reported drought-tolerance phenotypes.

The robustness and sensitivity of the WUE assay was demonstrated in Figure 6, which shows expected drought-intensity response phenotypes, with declining above-ground biomass, transpiration and water content at lower water levels. In contrast, there is no clear dose response to acetic acid concentration, but rather plants watered with 10–29 mM [HAc] + [Ac^–^] in potting mix had reduced growth and water use, compared to both lowest (0 mM) and highest (47 mM) treatments. Intriguingly, a reduction in transpiration rate in response to 29 mM [HAc] + [Ac^–^] (Figure 6) aligned closely with improved desiccation survival in maize treated with 30 mM previously observed (Kim et al., 2017), which could be expected from such anti-transpirant behavior. However, this reduction in water use was not associated with any phenotypes associated with improved performance under drought, such as WUE or biomass (Figure 6). Furthermore, as with Kim et al. (2017) these phenotypes disappeared at both lower and higher acetic acid doses (Figure 6). Precise concentration control in crops would be agronomically challenging for such a substrate for microbial growth (Sigren et al., 1997) with high solubility. Field experiments in soil under agronomically-relevant limited water availability are required to determine, first, whether acetic acid-induced reductions in water use and root growth are reproducible, and second, if this is associated with an increase in yield.

## Conclusion

The concentration of the membrane permeable undissociated form of acetic acid was demonstrated to drive maize seedling root inhibition under unbuffered conditions. This was confirmed in potting media, although peat provided partial protection from high [HAc]. A reduction in transpiration was observed with 29 mM [HAc] + [Ac^–^], but this did not lead to an increase in growth or interaction with deficit irrigation. Field trials are necessary to determine the biostimulant potential of this reduction in water use, under agronomically-relevant water-limited conditions. Furthermore, future studies on acetic acid impacts on drought tolerance need to characterize the treatment in terms of [HAc], and root growth inhibition impacts on transpiration should be excluded before claims of improved performance with reduced water availability are warranted.

## Data Availability Statement

All datasets generated for this study are included in the article/supplementary material.

## Author Contributions

MA and DA designed the study. MA completed the experiments. DA conceived the study, performed the statistical analysis and wrote the first draft of the manuscript. All authors contributed to manuscript revision, read and approved the submitted version.

## Conflict of Interest

DA was an employee of Shell International Exploration and Production Inc.

The remaining author declares that the research was conducted in the absence of any commercial or financial relationships that could be construed as a potential conflict of interest.
